# Supplementary figures and images for: Genome wide DNA methylation profiling identifies specific epigenetic features in high-risk cutaneous squamous cell carcinoma
Source: PLoS One. 2019 Dec 20;14(12):e0223341. doi: 10.1371/journal.pone.0223341 (PMC6924689; doi:10.1371/journal.pone.0223341)

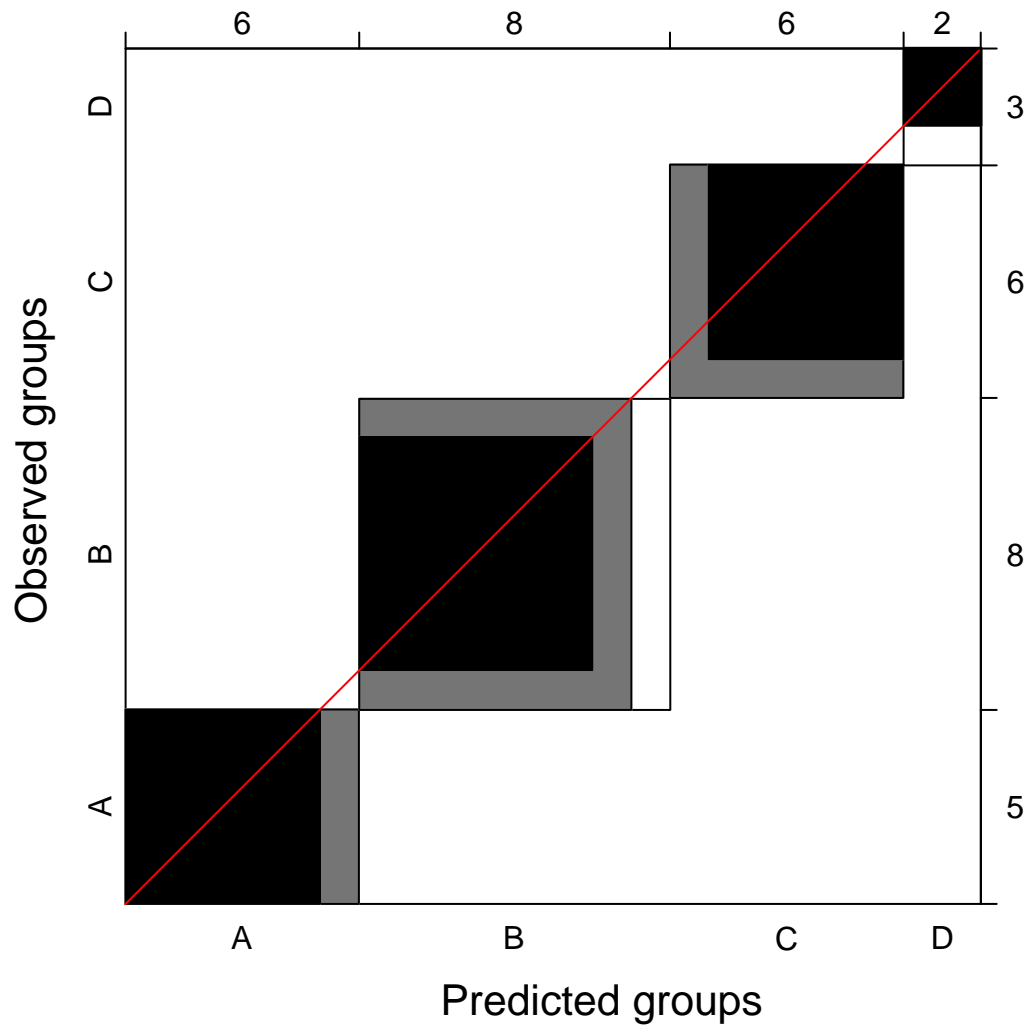

Supplement: S1 Fig — Graph represents the agreement plot derived from providing visual assessment of agreement between observed and predicted values from pyrosequencing data of 5CpG in our cohort as described in Bangdiwala et al. 2008, Journal of Clinical Epidemiology 61:866–874. Shaded areas represent cell frequencies from the confusion matrix of the adjusted multinomial model. The degree of agreement is visually expressed by the proportion of area in the darkened squares compared with the total area defined by the row and column marginal totals. A: actinic keratosis; B: low-risk cSCC; C: high-risk non metastatic cSCC; D: high-risk metastatic cSCC. Note that only 22 out of the 23 samples described in the manuscript could be analysed in the pyrosequencing assay. (PDF) [file pone.0223341.s001.pdf]
