# Supplementary material for: Genome wide DNA methylation profiling identifies specific epigenetic features in high-risk cutaneous squamous cell carcinoma
Source: PLoS One. 2019 Dec 20;14(12):e0223341. doi: 10.1371/journal.pone.0223341 (PMC6924689; doi:10.1371/journal.pone.0223341)
Supplement: S1 Table — (DOCX) [file pone.0223341.s002.docx]

**Table S1.** Patient samples.

| **Sample** | **Type of lesion** | **Date of diagnosis** | **Age at diagnosis** | **Time of evolution (months)** | **Location** | **Size (mm)** | **TNM stage at diagnosis (AJCC-8)** | **Histological differentiation** | **Survival (days)** | **Cause of death** |
| --- | --- | --- | --- | --- | --- | --- | --- | --- | --- | --- |
| 1 | ACTINIC KERATOSIS | 12/12/16 | 81 |  | SCALP |  | na | KIN III |  |  |
| 2 | ACTINIC KERATOSIS | 21/11/16 | 78 |  | SCALP |  | na | KIN II |  |  |
| 3 | ACTINIC KERATOSIS | 22/11/16 | 75 |  | SCALP |  | na | KIN I |  |  |
| 4 | ACTINIC KERATOSIS | 15/11/16 | 75 |  | SCALP |  | na | KIN II |  |  |
| 5 | ACTINIC KERATOSIS | 27/11/16 | 88 |  | SCALP |  | na | KIN II |  |  |
| 6 | LOW-RISK INITIAL INVASIVE | 7/1/15 | 93 | 4 | RIGHT CHEEK | 65 | T1N0M0 | moderately differentiated cSCC |  |  |
| 7 | LOW-RISK INITIAL INVASIVE | 15/1/15 | 87 | 6 | SCALP | 20 | T2N0M0 | poorly differentiated cSCC | 590 | Loco-regional progress N+ |
| 8 | LOW-RISK INITIAL INVASIVE | 16/12/15 | 85 | 3 | SCALP | 35 | T2N0M0 | poorly differentiated cSCC |  |  |
| 9 | LOW-RISK INITIAL INVASIVE | 27/11/14 | 72 | 5 | CUTANEOUS LIP | 20 | T1N0M0 | moderately differentiated cSCC |  |  |
| 10 | LOW-RISK INITIAL INVASIVE | 24/9/15 | 80 | 6 | RIGHT TEMPLE | 25 | T2N0M0 | moderately differentiated cSCC | 1190 | Local progression skull base |
| 11 | LOW-RISK INITIAL INVASIVE | 10/11/15 | 75 | 3 | CUTANEOUS LIP | 12 | T1N0M0 | well-differentiated cSCC |  |  |
| 12 | LOW-RISK INITIAL INVASIVE | 29/9/15 | 66 | 6 | LEFT TEMPLE | 24 | T1N0M0 | poorly differentiated cSCC | 721 | Loco-regional progress N+ |
| 13 | LOW-RISK INITIAL INVASIVE | 14/10/15 | 80 | 3 | LEFT TEMPLE | 12 | T2N0M0 | well-differentiated cSCC |  |  |
| 14 | LOW-RISK INITIAL INVASIVE | 15/7/15 | 80 | 6 | RIGHT TEMPLE | 10 | T1N0M0 | well-differentiated cSCC |  |  |
| 15 | LOW-RISK INITIAL INVASIVE | 22/8/16 | 73 | 4 | RIGHT TEMPLE | 25 | T1N0M0 | well-differentiated cSCC |  |  |
| 16 | HIGH-RISK NON METASTATIC | 15/1/16 | 70 | 8 | FOREHEAD | 60 | T3N0M0 | moderately differentiated cSCC | 182 | Bone and Lung M+ |
| 17 | HIGH-RISK NON METASTATIC | 15/11/15 | 73 | 4 | SCALP | 30 | T3N0M0 | moderately differentiated cSCC |  |  |
| 18 | HIGH-RISK NON METASTATIC | 30/6/16 | 76 | 3 | FOREHEAD | 50 | T3N0M0 | well-differentiated cSCC |  |  |
| 19 | HIGH-RISK NON METASTATIC | 20/4/16 | 75 | 2 | RIGHT CHEEK | 35 | T3N0M0 | moderately differentiated cSCC | 235 | Local progression skull base |
| 20 | HIGH-RISK NON METASTATIC | 1/10/15 | 63 | 50 | THORAX | 300 | T3N0M0 | well-differentiated cSCC | 680 | Local progression thorax |
| 21 | HIGH-RISK METASTATIC | 12/5/16 | 90 | 8 | INNER CANTHUS | 50 | T3N1M0 | moderately differentiated cSCC |  |  |
| 22 | HIGH-RISK METASTATIC | 13/10/15 | 45 | 6 | LEFT MALAR | 60 | T3N1M0 | well-differentiated cSCC |  |  |
| 23 | HIGH-RISK METASTATIC | 13/4/14 | 82 | 4 | RIGHT TEMPLE | 20 | T3N1M0 | moderately differentiated cSCC | 471 | Loco-regional progress N+ |
