# Supplementary material for: Genome wide DNA methylation profiling identifies specific epigenetic features in high-risk cutaneous squamous cell carcinoma
Source: PLoS One. 2019 Dec 20;14(12):e0223341. doi: 10.1371/journal.pone.0223341 (PMC6924689; doi:10.1371/journal.pone.0223341)
Supplement: S2 Table — (DOCX) [file pone.0223341.s003.docx]

Differentially methylated CpGs between groups

| **p-value** | **No of DM CpG** |
| --- | --- |
| 0.05 | 272964 |
| 0.01 | 164842 |
| 0.001 | 92371 |
| 10^-4^ | 56969 |
| 10^-5^ | 37339 |
| 10^-6^ | 25260 |
| 10^-7^ | 17293 |
| 10^-8^ | 11716 |
| 10^-9^ | 8002 |
| 10^-10^ | 5354 |
