# Supplementary material for: Genome wide DNA methylation profiling identifies specific epigenetic features in high-risk cutaneous squamous cell carcinoma
Source: PLoS One. 2019 Dec 20;14(12):e0223341. doi: 10.1371/journal.pone.0223341 (PMC6924689; doi:10.1371/journal.pone.0223341)
Supplement: S6 Table — (DOCX) [file pone.0223341.s007.docx]

**Table S6.** Enriched KEGG pathways

| **Enrichment FDR** | **Genes in list** | **Total genes** | **Functional Category** | **Genes** |
| --- | --- | --- | --- | --- |
| 0.001451203 | 10 | 98 | Phosphatidylinositol signaling system | INPP5F PLCB1 INPP5A ITPR1 PRKCB ITPKC PIP5K1B SYNJ2 PLCZ1 MTMR7 |
| 0.002327198 | 8 | 73 | Inositol phosphate metabolism | INPP5F PLCB1 INPP5A ITPKC PIP5K1B SYNJ2 PLCZ1 MTMR7 |
| 0.002327198 | 10 | 116 | Thyroid hormone signaling pathway | MED12L CREBBP PLCB1 NOTCH1 PRKCB MAP2K2 RXRA SLC2A1 SLC9A1 PLCZ1 |
| 0.003125413 | 11 | 151 | Oxytocin signaling pathway | ADCY9 PLCB1 PIK3R5 ITPR1 NFATC1 PRKAG2 PRKCB MAP2K2 CACNA1C CAMK2B CACNA2D4 |
| 0.005249892 | 7 | 66 | Long-term potentiation | CREBBP PLCB1 ITPR1 PRKCB MAP2K2 CACNA1C CAMK2B |
| 0.007111117 | 9 | 119 | Vascular smooth muscle contraction | MRVI1 ADCY9 PLCB1 ITPR1 PRKCB MAP2K2 CACNA1C CALD1 PLA2G6 |
| 0.021463974 | 10 | 180 | Calcium signaling pathway | ADCY9 DRD1 PLCB1 ITPR1 PDE1C PRKCB CACNA1C ITPKC CAMK2B PLCZ1 |
| 0.021463974 | 5 | 48 | Notch signaling pathway | CREBBP NOTCH1 MAML3 PSEN1 NCOR2 |
| 0.021463974 | 5 | 47 | Hedgehog signaling pathway | GPR161 ARRB2 SUFU SMURF2 SPOP |
| 0.021463974 | 7 | 96 | Inflammatory mediator regulation of TRP channels | ADCY9 CYP2J2 PLCB1 ITPR1 PRKCB CAMK2B PLA2G6 |
| 0.021463974 | 7 | 92 | GnRH signaling pathway | ADCY9 PLCB1 ITPR1 PRKCB MAP2K2 CACNA1C CAMK2B |
| 0.021463974 | 6 | 69 | Adipocytokine signaling pathway | NFKB1 PRKAG2 ACSL5 RXRA SLC2A1 TRADD |
| 0.021739776 | 7 | 100 | HIF-1 signaling pathway | CREBBP HK1 NFKB1 PRKCB MAP2K2 SLC2A1 CAMK2B |
| 0.021739776 | 7 | 100 | Melanogenesis | ADCY9 CREBBP PLCB1 PRKCB MAP2K2 WNT5B CAMK2B |
| 0.021739776 | 6 | 74 | Gastric acid secretion | ADCY9 PLCB1 ITPR1 PRKCB SLC9A1 CAMK2B |
| 0.022135307 | 13 | 295 | MAPK signaling pathway | TAB1 DUSP3 FGFR1 ARRB2 NFATC1 NFKB1 PRKCB MAP2K2 CACNA1C DUSP16 TRADD MAPKAPK2 CACNA2D4 |
| 0.022135307 | 9 | 161 | CGMP-PKG signaling pathway | MRVI1 ADCY9 PLCB1 PIK3R5 ITPR1 NFATC1 MAP2K2 VASP CACNA1C |
| 0.024991459 | 8 | 136 | Apelin signaling pathway | ADCY9 PLCB1 PIK3R5 ITPR1 PRKAG2 MAP2K2 SLC9A1 HDAC4 |
| 0.024991459 | 7 | 106 | Parathyroid hormone synthesis, secretion and action | ADCY9 FGFR1 PLCB1 ITPR1 ARRB2 PRKCB RXRA |
| 0.028490721 | 6 | 83 | TGF-beta signaling pathway | CREBBP LTBP1 SMURF2 TFDP1 TGIF1 ACVR1B |
| 0.028490721 | 9 | 175 | Axon guidance | SEMA6B SEMA4F LIMK2 SEMA5B SLIT1 WNT5B CAMK2B NCK2 UNC5C |
| 0.028490721 | 7 | 112 | Cholinergic synapse | ADCY9 PLCB1 PIK3R5 ITPR1 PRKCB CACNA1C CAMK2B |
| 0.028490721 | 6 | 85 | Insulin secretion | ADCY9 PLCB1 PRKCB SLC2A1 CACNA1C CAMK2B |
| 0.030517508 | 8 | 146 | Wnt signaling pathway | CREBBP PLCB1 NFATC1 PPARD PRKCB PSEN1 WNT5B CAMK2B |
| 0.031103033 | 6 | 88 | Gap junction | ADCY9 DRD1 PLCB1 ITPR1 PRKCB MAP2K2 |
| 0.032292009 | 35 | 1274 | Metabolic pathways | MGAT4A MGLL CYP2J2 ALDH1A3 INPP5F LPIN1 PLCB1 PISD GALNS GLB1 HAL HDC HK1 INPP5A CYP4F3 OGDH PCYT1A ACSL5 PGD NADSYN1 CHDH POLR3B PRODH GALNT16 NADK TBXAS1 ITPKC PIP5K1B PLA2G6 GPT2 SYNJ2 PLCZ1 MTMR7 PIGS PGS1 |
| 0.032292009 | 6 | 91 | Fc gamma R-mediated phagocytosis | GSN LIMK2 PRKCB VASP PIP5K1B PLA2G6 |
| 0.032292009 | 9 | 185 | Kaposi s sarcoma-associated herpesvirus infection | CREBBP PIK3R5 ITPR1 NFATC1 NFKB1 MAP2K2 TYK2 TRADD MAPKAPK2 |
| 0.032292009 | 18 | 523 | Pathways in cancer | LAMC3 ADCY9 CREBBP CSF2RB FGFR1 PLCB1 NFKB1 NOTCH1 SUFU PPARD PRKCB MAP2K2 LPAR5 RXRA SLC2A1 WNT5B CAMK2B RUNX1 |
| 0.032424914 | 5 | 65 | Central carbon metabolism in cancer | FGFR1 HK1 MAP2K2 SLC2A1 SLC16A3 |
