# Supplementary material for: Genome wide DNA methylation profiling identifies specific epigenetic features in high-risk cutaneous squamous cell carcinoma
Source: PLoS One. 2019 Dec 20;14(12):e0223341. doi: 10.1371/journal.pone.0223341 (PMC6924689; doi:10.1371/journal.pone.0223341)
Supplement: Attachment — Submitted filename: Response to Reviewers.pdf [file pone.0223341.s009.pdf]

RESPONSE TO REVIEWERS

PONE-D-19-25692
Genome wide DNA methylation profiling identifies specific epigenetic features in high-risk cutaneous squamous cell carcinoma.

Response to Reviewer 1

We agree with the reviewer's comment regarding the limited number of samples included in the study. Obviously, in this kind of studies the more samples the better. However, we would like to emphasize that other cSCC studies with a similar strategy, that is, the use of epigenomics arrays, used a comparable number of samples. *Vandiver et al* ***Genome Biol****. 2015;16:80* analyzed 7 cSCC samples, whereas *Rodriguez-Paredes M et al.* ***Nat Commun****. 2018;9(1):577* characterized 16 actinic keratosis samples against 18 cSCC samples. Moreover, none of these studies classify samples in four different stages, which, as the Reviewer kindly comment, is the strength of our study.

Major comments have been addressed as follow:

1. What is the bisulfite conversion efficiency for the EPIC beachip? Are they similar among 4 groups?
As a part of our standard protocol, we checked the bisulfite conversion reactions analyzing the BS Conversion I and BS Conversion II control probes as described in Illumina user guide. In all the samples, the efficiency of bisulfite conversion was optimal with no significant differences among sample groups. We have introduced a sentence in the Methods section (page 6) to clarify this point.

2. A large number of differentially methylated CpG sites was identified among four groups. The cellularity for these samples is not presented in the manuscript, so it is difficult to evaluate if different carcinoma contents potentially confound this analysis.

It was mentioned in the Materials and Methods section, at the end of Patient samples block, that *'All samples were evaluated visually by a trained dermatopathologist to validate tumour cellularity'*. Indeed, tumour cells in most of the samples represent around a 80%-90% of total cells, and no bias between groups was observed. This data is now included in the text (page 5) to avoid confusion.

3. The author reported that there are no sequential DNA methylation changes occur in the development of sSCC. Only 23 samples were included in the analysis. Is this sample size adequate to draw this conclusion?

We agree with the reviewer that the statistical power is not enough to rule out the existence of specific and individual CpGs sequential changes. However, our ordinal regression analysis, which assumed changes to be sequential from actinic keratosis to high-risk metastatic cSCC, did not find any CpG able to discriminate among groups. In contrast, the multinomial regression analysis, which was not restricted by the sequential effects assumption, found clear methylation patterns discriminating among the four groups. Considering the reviewer's suggestion, we have softened the statement regarding the sequential methylation changes. Now it is stated in page 9 that: *'This indicates that no evident sequential DNA methylation changes occur in the development of cSCC.'*

4. Considering only 94 selected CpG sites, the authors found that the methylation level is lower in the initial invasive group than premalignant actinic keratosis, while it is higher in the high-risk groups compared to the low-risk group. Does this trend also true for the global methylation level?

As stated in the previous response, the statistical power is not large enough to draw strong global conclusions regarding methylation patterns, in contrast to the 94 CpG signature which clearly discriminates between the four groups and clearly shows the mentioned pattern. Nevertheless, when examining random subsamples of the whole dataset in a heatmap (Figure 1B), the pattern showed always this same behavior. This is in fact stated in the text in the first paragraph of page 10.

5. A prognostic prediction model was established considering all CpGs as potential predictors. Again, the sample size is a limitation. The rational for establishing the prediction model is unclear. It is highly possible that the prognostic value of this signature comes from the association with stage. If it is the case, then, measurement of methylation biomarkers with additional cost does not add any clinical value in practice.

In this approach we wanted to emphasize the potential role of epigenomics not only in diagnosis but also in prognosis. We agree with the reviewer that the sample size is a limitation and further validation of this model should be performed in future studies. Nevertheless, the model was fitted with an advanced statistical method, elastic net, which is specifically suited for dealing with data sets with a large number of variables and very few observations. Regarding the possibility of an association with stage, we assessed the capability of each of the included CpGs in the model in discriminating between the four stages. Remarkably, no CpG showed discriminative power among stages, with the lowest p-value being 0.24. Also, a cox regression model including stage as predictor showed an AUC of ‘only’ 0.72, compared to the AUC of 0.98 of our model including 32 CpGs. Therefore, no evidence suggests that this epigenetic signature might be confounded by stage, which reinforces its value as a potential biomarker for survival prognosis.

RESPONSE TO REVIEWERS

PONE-D-19-25692
Genome wide DNA methylation profiling identifies specific epigenetic features in high-risk cutaneous squamous cell carcinoma.

Response to Reviewer 2

1. The methylation analysis showed that the low-risk invasive cSCC exhibited lower methylation levels than premalignant AK, and high-risk non-metastatic and metastatic cSCC exhibited higher methylation levels, indicating a non-sequential and complex pattern of DNA-methylation during cSCC evolution. This is an interesting finding, the authors would be advised to discuss the possible cause of this changes.
It is not obvious the significance of this non-sequential pattern of DNA-methylation during cSCC evolution. What it is clear when the comparison is restricted to low-risk against high-risk samples, is that malignancy is associated to a higher DNA-methylation level. It is tempting to speculate that evolution from premalignant actinic keratosis could follow alternative pathways to evolve to a low-risk stage when DNA-methylation is lowered or a high-risk stage when DNA-methylation is increased. In fact, clinical analysis of patients reveals that not all high-risk cSCC have necessarily passed through a low-risk stage. This hypothesis is now commented in the Discussion section in page 14.

2. All of the data are based on microarray analysis, it is better to select several genes for MSP or pyrophosphate sequencing validation, which would support their findings and conclusions.

We have carried out a pyrophosphate sequencing with 5 CpG of the 94 CpG signature discriminating between the four different stages. The results validated the discrimination power of the signature, since the pyrosequencing data was able to correctly classify 82% of the samples (18 out of 22) by using only 5 CpGs out of the 94 CpGs from the original signature and achieved a weighted Bangdiwala score of 0.90 out of 1 in the agreement test (*Bangdiwala et al 2008,The agreement chart as an alternative to the receiver-operating characteristic curve for diagnostic tests. Journal of Clinical Epidemiology, 61 (9), 866-874*). These results are now commented in the Results section in page 11 and the representation of the confusion matrix as an agreement plot is included as Supplementary Figure 1.
