# Supplementary material for: Genome wide DNA methylation profiling identifies specific epigenetic features in high-risk cutaneous squamous cell carcinoma
Source: PLoS One. 2019 Dec 20;14(12):e0223341. doi: 10.1371/journal.pone.0223341 (PMC6924689; doi:10.1371/journal.pone.0223341)
Supplement: Attachment — Submitted filename: Response to Reviewer.pdf [file pone.0223341.s010.pdf]

RESPONSE TO REVIEWER

PONE-D-19-25692
Genome wide DNA methylation profiling identifies specific epigenetic features in high-risk cutaneous squamous cell carcinoma.

Response to Reviewer 1

We are extending the response regarding Reviewer’s concern about bisulfite conversion since it seems it was not clearly answered. As we stated in our previous response, we checked our Illumina´s internal controls as a routine step in standard protocols in methylation analysis. The analysis of BS Conversion I and BS Conversion II control probes were in the standard parameters recommended by Illumina user guide. We provide now the graphs with this information from Illumina examples and from our experiment to be checked by the Reviewer. We hope this information helps to clarify this point. We would like to add that unless new or modified Illumina protocols are described in the studies, these Illumina´s internal controls are not usually included in manuscripts. Therefore, we have not included them in our manuscript, although we can do it if the Reviewer considers it is necessary.

This is the text that *GenomeStudio® Methylation Module v1.8 User Guide* indicates for bisulfite conversion controls. We would like to clarify that Illumina did not update this guide when the Infinium EPIC DNA methylation beadchip array used in this study was released. Therefore, controls in our array are quite similar but not identical to the showed in the user guide.

HumanMethylation450

Bisulfite Conversion I

These controls use Infinium I probe design and allele-specific single base extension to monitor efficiency of bisulfite conversion. If the bisulfite conversion reaction was successful, the "C" (Converted) probes will match the converted sequence and get extended. If the sample has unconverted DNA, the "U" (Unconverted) probes will get extended. There are no underlying C bases in the primer landing sites, except for the query site itself. Performance of bisulfite conversion controls C1, C2 and C3 should be monitored in the Green channel, and controls C4, C5 and C6 should be monitored in Red channel.

Figure 55 Bisulfite Conversion I Controls

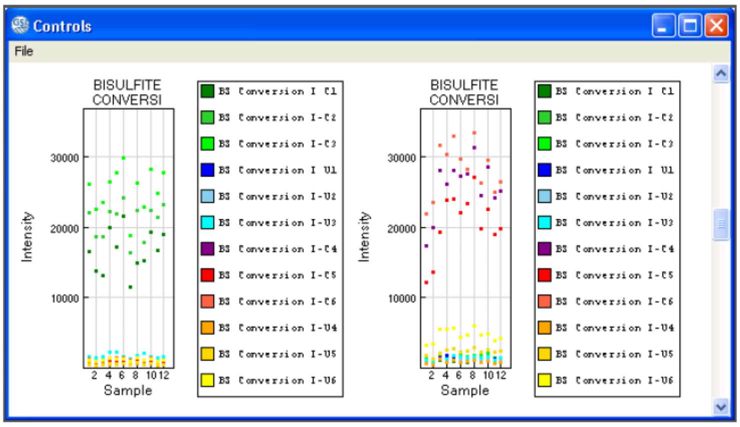

Bisulfite Conversion II

These controls use Infinium II probe design and single base extension to monitor efficiency of bisulfite conversion. If the bisulfite conversion reaction was successful, the "A" base will get incorporated and the probe will have intensity in the Red channel. If the sample has unconverted DNA, the "G" base will get incorporated across the unconverted cytosine, and the probe will have elevated signal in the Green channel.

Figure 56 Bisulfite Conversion II Controls

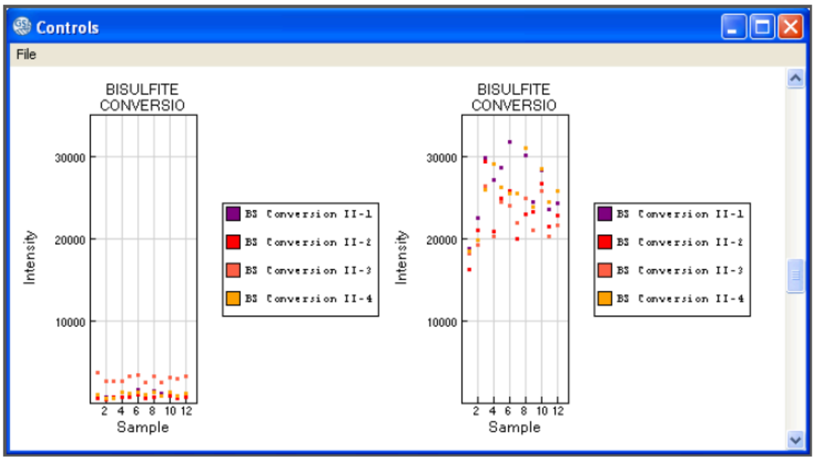

And these are the results for our samples:

Bisulfite Conversion I controls

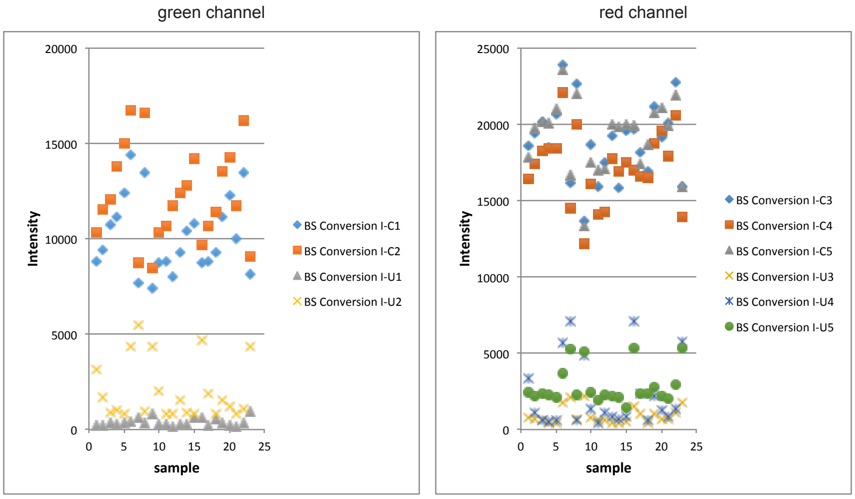

Bisulfite Conversion II controls

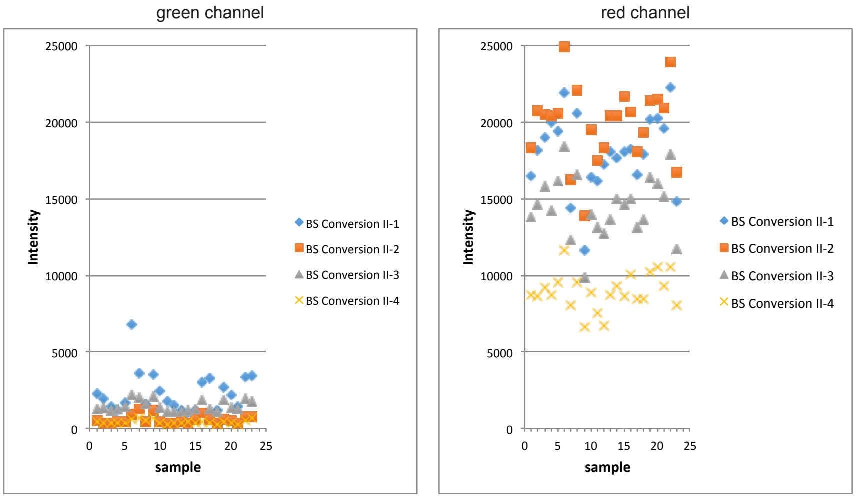

Thanks for indicating the presence of grammatical errors. A native English speaker author had edited writing and now another independent native English speaker has edited it.
